# Therapeutic potential of bone marrow-derived mesenchymal stem cells for cutaneous wound healing

**DOI:** 10.3389/fimmu.2012.00192

**Published:** 2012-07-10

**Authors:** Jerry S. Chen, Victor W. Wong, Geoffrey C. Gurtner

**Affiliations:** Department of Surgery, Stanford UniversityStanford, CA, USA

**Keywords:** mesenchymal stem cells, wound healing, differentiation, paracrine signaling, tissue engineering

## Abstract

Despite advances in wound care, many wounds never heal and become chronic problems that result in significant morbidity and mortality to the patient. Cellular therapy for cutaneous wounds has recently come under investigation as a potential treatment modality for impaired wound healing. Bone marrow-derived mesenchymal stem cells (MSCs) are a promising source of adult progenitor cells for cytotherapy as they are easy to isolate and expand and have been shown to differentiate into various cell lineages. Early studies have demonstrated that MSCs may enhance epithelialization, granulation tissue formation, and neovascularization resulting in accelerated wound closure. It is currently unclear if these effects are mediated through cellular differentiation or by secretion of cytokines and growth factors. This review discusses the proposed biological contributions of MSCs to cutaneous repair and their clinical potential in cell-based therapies.

## Introduction

Chronic wounds are a cause of significant morbidity and mortality and pose a large financial burden on the healthcare system. Proper cutaneous wound repair requires a well-coordinated response of inflammation, neovascularization, extracellular matrix formation, and epithelialization. Failure of any of these processes due to ischemia, reperfusion injury, bacterial infection, or aging can result in chronic inflammation and a non-healing wound (Mustoe et al., 2006).

Traditional therapies for the treatment of chronic wounds include debridement, minimization of bacterial load, pressure offloading, negative-pressure therapy, biological dressings, skin grafting, and reconstructive tissue flaps. Despite the most recent advances in wound management, up to 50% of chronic wounds still fail to heal (Cha and Falanga, 2007). One hypothesis for this problem is that resident cells in non-healing wounds are intrinsically impaired and demonstrate increased senescence and decreased response to growth factors (Hasan et al., 1997; Vande Berg et al., 1998).

Bone marrow-derived mesenchymal stem cells (MSCs) were originally described as plastic-adherent fibroblast-like cells and can differentiate into osteoblasts, adipocytes, and chondrocytes (Friedenstein et al., 1976; Pittenger et al., 1999). Their plasticity has since been expanded to include contribution to cell lineages in brain (Brazelton et al., 2000), muscle (Ferrari et al., 1998), liver (Alison et al., 2000), and kidney tissue (Poulsom et al., 2001). MSCs are easy to isolate and expand in culture and studies have suggested minimal immunogenic response when allogeneic or syngeneic cells are used (Ryan et al., 2005; Chen et al., 2009). Given these qualities and the current barriers limiting embryonic stem cell research, MSCs have become a recent focus of interest for cellular therapy in tissue regeneration. The application of MSCs for tissue repair has ranged from intravenous infusion to reduce the size of brain infarcts in a rat stroke model (Li et al., 2005) to implantation of cells in the myocardium to reduce left ventricular dysfunction in a swine model of myocardial ischemia (Amado et al., 2005). Here we review the existing evidence for MSC-based therapies for cutaneous wound healing and future directions to bring their potential to the clinical setting.

## Bone marrow-derived cells in normal post-natal skin development

Early literature demonstrating the contribution of bone-marrow derived cells to the epidermis formed the basis for investigating the role for BM-MSCs specifically in cutaneous repair. The development of transgenic mice strains expressing green fluorescent protein (GFP) has been essential in allowing investigators to understand the behavior of cells *in vivo*. When coupled with bone marrow transplantation or parabiosis models, GFP+ donor cells can be identified in wild-type mice using basic immunohistochemistry techniques to track cellular fate and differentiation. The ability for precursor cells to mobilize from the bone marrow niche to peripheral tissue remains controversial, however, several investigators have demonstrated the existence of circulating bone marrow precursor cell (Roufosse et al., 2004). Several early animal studies have reported that in normal skin homeostasis, bone marrow-derived cells may contribute to keratinocytes in the epidermis and sebaceous glands as well as dendritic cells within the dermis. The aggregate contribution to the epidermis and dermis by cells of bone marrow origin has been described as 11–14% of the total cell population (Fathke et al., 2004; Deng et al., 2005). Cell fusion between bone marrow-derived cells and mature resident cells has been observed in *in vitro* co-culture systems by several groups resulting in cells that adapt a “differentiated” phenotype but fail to undergo true differentiation (Terada et al., 2002; Spees et al., 2003). Several investigators have attempted to address this phenomenon and have demonstrated a lack of cell fusion in these models by using sex-mismatched donor cells and performing FISH analysis (Brittan et al., 2005; Wu et al., 2007; Sasaki et al., 2008). In a similar model, Badiavas et al. utilized a total bone marrow transplantation model and discovered bone marrow-derived CD34+ (a hematopoietic stem cell marker) keratinocytes in the hair bulge region which is thought to be the stem cell niche for epidermal stem cells (Badiavas et al., 2003; Trempus et al., 2003). This suggests that potentially circulating bone marrow-derived cells may serve to replenish the epithelial stem cell compartment throughout life. While still early, these studies highlight the potential role of bone marrow-derived stem cells in differentiating into various lineages to maintain skin homeostasis.

## MSC delivery enhances cutaneous wound repair

Wound healing studies have subsequently focused on MSCs as the potential cell population within bone marrow that can contribute to cutaneous regeneration. Studies in both mice and humans have consistently demonstrated enhanced wound repair following treatment with bone marrow-derived MSCs (Table 1).

**Table 1:** **Study design and results for treatment of cutaneous wounds with mesenchymal stem cell therapy**.

**Species**	**Wound type**	**Therapy type**	**Delivery method**	**Control(s)**	**Findings**	**Reference**
Mouse	Excisional wounds	Concentrated conditioned medium from allogeneic P3 MSCs	Single subcutaneous injection of 80 ul and topical application of 20 ul conditioned medium	Concentrated conditioned medium from dermal fibroblasts	Accelerated wound closure. Increased recruitment of macrophages and endothelial progenitor cells	Chen et al., 2008
Mouse	Excisional wounds	Allogeneic P3 MSCs	Single systemic injection of 1 × 10^6^ cells	PBS	Accelerated wound healing	Sasaki et al., 2008
Mouse	Excisional wounds	Allogeneic P3-6 MSCs	Topical application of 2.5 × 10^5^ cells seeded on hydrogel scaffold	No treatment, hydrogen alone, and intradermal injection	Accelerated wound healing. Increased angiogenesis and restoration of hair follicles and sebaceous glands.	Rustad et al., 2012
Mouse (wild type and diabetic)	Excisional wounds	Allogeneic P3-5 MSCs	Single intradermal injection of 1 × 10^6^ cells	Dermal fibroblasts	Accelerated wound closure. Increased granulation tissue, angiogenesis, and restoration of hair follicles.	Wu et al., 2007
Mouse (diabetic)	Excisional wounds	Allogeneic P33 MSCs	Single topical application of 7.5 × 10^5^ cells	PBS	Accelerated wound closure. Increased granulation tissue and angiogenesis.	Javazon et al., 2007
Rat	Incisional fascial wounds	Allogeneic P3-5 MSCs	Four systemic injections of 2 × 10^6^ cells OR. Single intradermal injection of 6 × 10^6^ cells	PBS	Increased wound burst strength. Increased collagen composition	McFarlin et al., 2006
Rat (diabetic)	Incisional fascial wounds	Allogeneic P2-6 MSCs	Four systemic injections of 1.5 × 10^6^ cells OR. Single intradermal injection of 6 × 10^6^ cells	PBS	Increased wound burst strength. Increased collagen composition	Kwon et al., 2008
Human	Chronic non-healing wounds (*n* = 3)	Autologous bone marrow aspirate and cultured MSCs	Subcutaneous injection of bone marrow aspirate and 1–3 topical applications of MSCs	None	Complete closure of wounds. Increased inflammatory response and angiogenesis.	Badiavas and Falanga, 2003
Human	Chronic non-healing wounds (*n* = 24)	Autologous P0 MSCs	Intramuscular and subcutaneous injection of > 1 × 10^6^ cells/cm^2^ ulcer area and topical application	Standard wound care	Decreased wound size. Increased pain-free walking distance	Dash et al., 2009
Human	Acute (*n* = 4) and chronic (*n* = 6) non-healing wounds	Autologous P2-10 MSCs	1–3 topical applications by fibrin spray	None	Complete healing of acute wounds. Reduction or complete closure of chronic wounds. Dose dependent effect.	Falanga et al., 2007
Human	Chronic non-healing wounds (*n* = 20)	Autologous P0 MSCs	Topical application of MSC seeded collagen sponge	None	Complete closure of 13 wounds. Partial closure of five wounds.	Yoshikawa et al., 2008

MSC, mesenchymal stem cell; P, passage.

The use of murine models has been crucial for advancing the understating of wound healing, however, fundamental differences exist between the mouse and human skin. Murine skin lacks apocrine sweat glands and rete ridges/dermal papillae, which are both found in human skin. However, rete ridge-like structures may become apparent during mouse wound healing and are often described as “pseudoepitheliomatous” or “pseudocarcinomatous hyperplasia” (Sundberg, 2004). Mouse skin also has a panniculosus carnosus layer, a thin subcutaneous muscle layer only found in the human neck (platysma). This muscle layer produces rapid wound contraction following injury which is the primary method of wound healing in the mouse as opposed to granulation tissue formation and re-epithelialization in humans. Mouse skin has also been shown to be thinner and more compliant than human skin (Aarabi et al., 2007). A more complete summary of murine wound healing models is reviewed here (Wong et al., 2011b).

Experiments with diabetic murine models have been particularly useful in assessing the clinical utility of MSCs in wound repair. Many non-healing ulcers are caused by diabetic pathology which has been shown to attenuate the recruitment of inflammatory cells and down-regulate expression of various growth factors (Falanga, 2005). Local delivery of MSCs significantly increased granulation tissue formation and decreased wound healing time in leptin receptor-deficient db/db diabetic mice compared to those treated with either PBS or non-cell type-specific bone marrow aspirate (Javazon et al., 2007). Analysis of the mechanical properties of treated wounds revealed that administration of MSCs not only accelerated wound closure but also enhanced wound repair quality, resulting in healed tissue with increased tensile strength. This effect is thought to be secondary to increased collagen composition within the healed tissue (McFarlin et al., 2006; Kwon et al., 2008). The mechanism for this observed increase in collagen secretion is currently under investigation.

Promising findings in animal models have led to a very limited number of small-scale human trials examining the effects of autologous MSCs on chronic wounds. Injection of primary bone marrow cells into the wound edge followed by topical application of cultured MSCs resulted in the complete closure of three chronic wounds which had failed traditional therapy including autologous skin grafting (Badiavas and Falanga, 2003). Hallmarks of the healing wounds were a massive influx of mature and immature inflammatory cells, increased vascularity, and increased dermal thickness. It must be noted that this study utilized the injection of whole bone marrow aspirate which includes a large and mixed population of hematopoietic stem cells and inflammatory cells.

Dash et al. conducted the only randomized controlled trial investigating the use of MSCs in 24 patients with non-healing lower extremity ulcers secondary to diabetes or vasculitis. Autologous MSCs expanded in culture were injected intramuscularly into the wound edges of the treatment group. Twelve weeks after implantation, ulcer size in the MSC-treated group decreased 73% while those receiving standard wound care only decreased 23%. In addition, subjects receiving MSC injections increased their pain-free walking distance 7.5-fold compared to 2.2-fold in the control group with no reported adverse effects (Dash et al., 2009). Increased numbers of mature immune cells in the dermis of wound biopsies in the treatment group suggest an augmented inflammatory response as a possible mechanism for enhanced repair.

## MSCs enhance wound healing by differentiation into epidermal cells

**Figure 1:**
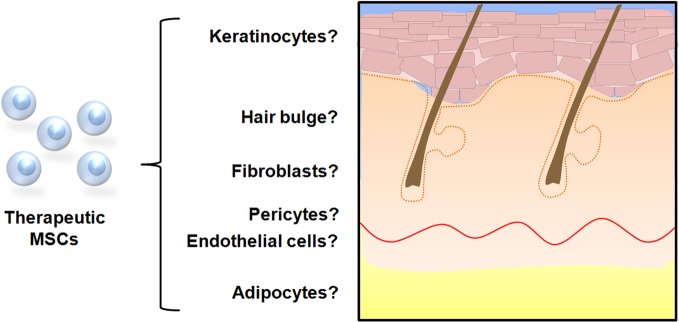
**Possible cell fate of mesenchymal stem cells in cutaneous repair.** *In vitro* studies have demonstrated that MSCs are capable of differentiating into cells with keratinocytic, fibroblastic, endothelial, and adipocytic phenotypes when cultured under specific conditions. Several studies utilizing wound healing models and transplanted labeled MSCs have provided evidence that cellular differentiation occurs *in vivo* to contribute to cutaneous repair.

There is data to suggest that MSCs mobilize from the bone marrow niche and traffic to ischemic tissue via the peripheral circulation in response to cytokine signaling (Hamou et al., 2009). Once at the site of injury, it is hypothesized that they contribute to wound healing by differentiating into various cells of the epidermis and dermis (Figure 1).

In culture, MSCs have been observed to differentiate into K14+ keratinocytes when grown in the presence of the growth factor BMP-4 (Sasaki et al., 2008). Histological examination of murine wounds treated with GFP + MSCs has demonstrated various levels of direct engraftment of donor cells into the epidermis as mature keratinocytes (Badiavas et al., 2003; Fathke et al., 2004; Harris et al., 2004). There is also evidence that transplanted MSCs are capable of ongoing differentiation as the percentage of GFP+ keratinocytes increases when wounds are analyzed over time. However, long-term engraftment has not been observed (Wu et al., 2007). Furthermore, differentiated cells have been observed to maintain active proliferation *in vivo* (Borue et al., 2004).

## MSCs enhance neovascularization during wound repair

New vessel formation, or neovascularization, is a critical component of wound healing as it is necessary to supply oxygen and nutrients to and carry waste away from the damaged tissue. Neovascularization can occur by two mechanisms: angiogenesis and vasculogenesis. During angiogenesis, tissue ischemia initiates signals that stimulate mature resident endothelial cells to proliferate and sprout new vessels (Folkman, 1995). In contrast, vasculogenesis involves the formation of de novo blood vessels from circulating vascular progenitor cells that home to the ischemic site (Tepper et al., 2005). *In vitro* experiments have demonstrated that MSCs are capable of differentiating into vessel forming endothelial cells suggesting that they may contribute to postnatal vasculogenesis during the wound healing process. When cultured in medium supplemented with VEGF, MSCs exhibit an endothelial-like phenotype such as expression of the vascular markers von Willebrand Factor (vWF), kinase insert domain receptor (KDR), and vascular cell adhesion molecule (VCAM). Furthermore, these MSCs form tube-like structures when cultured on Matrigel, an established *in vitro* model for neovessel formation (Oswald et al., 2004).

Similar evidence for endothelial differentiation has been extended to animal models. In a parabiosis model, GFP+ MSCs were found to traffic from the bone marrow to ischemic wounds and engraft into neovessels. Approximately 12% of all endothelial cells within the wound bed were determined to originate from donor MSCs (Hamou et al., 2009). Similar wound healing studies report the incidence of MSC-derived endothelial cells ranging from 0.1% to 13% (Badiavas et al., 2003; Fathke et al., 2004; Sasaki et al., 2008). MSC-treated excisional wounds in BALB/c mice demonstrated nearly two times the capillary density as quantified by CD31 staining than vehicle and fibroblast-treated wounds. In this study, engrafted MSCs were located in the perivascular space as opposed to the endothelium suggesting differentiation into pericytes. These cells do not directly incorporate into neovessels but are proposed to participate in angiogenesis by guiding endothelial sprouts (Nehls et al., 1992). Current research is elucidating how pericytes continue to support and regulate mature vessels through local secretion of soluble growth factors and mechanical signaling (Hirschi and D'Amore, 1997; Gerhardt and Betsholtz, 2003; Wu et al., 2007; Rustad et al., 2012).

## MSC-mediated paracrine signaling enhances wound repair

There is growing evidence to suggest that MSCs may elicit the majority of their wound healing properties via paracrine mechanisms. When compared to dermal fibroblasts, which are normally the main source of growth factors during cutaneous wound healing, MSCs express significantly greater amounts of VEGF-A, epidermal growth factor (EGF), erythropoietin, and stromal cell-derived factor -1α (SDF-1α) (Chen et al., 2008). The same group demonstrated that soluble proteins secreted by MSCs are potent mitogens. For example, keratinocytes and endothelial cells exhibit significantly greater proliferation rates when cultured in conditioned medium from MSCs compared to medium from fibroblast cultures. These proteins are also powerful chemoattractants and promote the migration of inflammatory cells, endothelial cells, and keratinocytes (Chen et al., 2008). Paracrine factors from MSCs have also been shown to stimulate collagen secretion from dermal fibroblasts *in vitro* (Kim et al., 2007). Excisional wounds treated with conditioned medium alone from MSC cultures demonstrate accelerated closure in wild-type mice, corroborating the importance of MSC-secreted factors in wound healing. These wounds also exhibited increased recruitment of macrophages, key cells in the acute healing process, and CD34+ and c-kit+ cells which have been described as the putative endothelial progenitor cell (Chen et al., 2008).

## Optimizing delivery of MSCs to cutaneous wounds

**Figure 2:**
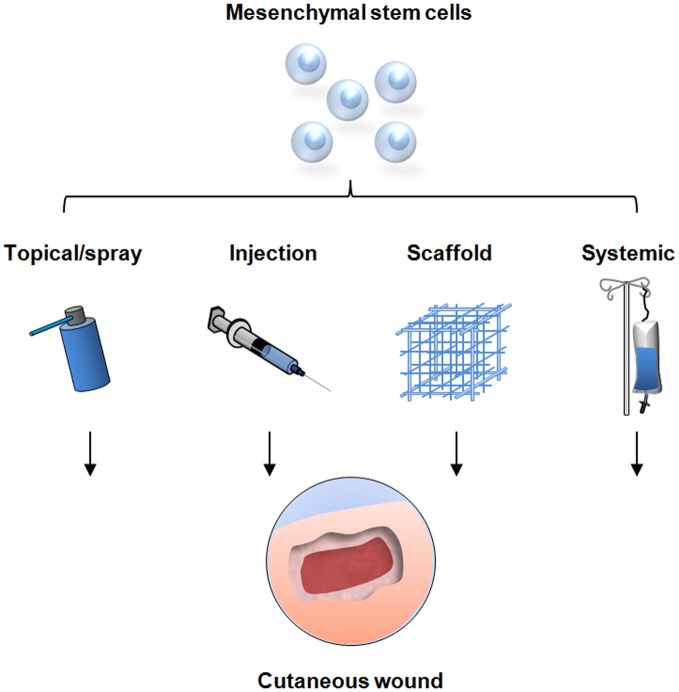
**Strategies for mesenchymal stem cell delivery to cutaneous wounds.** Traditional techniques include local injection of cells into the soft tissue, direct topical application, and systemic delivery via injection into the peripheral circulation. These methods have resulted in improved wound healing but are limited by sub-optimal cell survival and engraftment. Novel delivery methods are being developed utilizing tissue scaffolds to optimize stem cell function and maximize the therapeutic potential for cellular therapy.

As evidence for the wound healing capacity of MSCs continues to grow, research has now shifted toward modalities to optimize cell delivery as studies have shown that the clinical effectiveness of MSC-therapy is dependent on the number of cells delivered (Falanga et al., 2007). Most studies have utilized the technically simple method of injecting a cell suspension intradermally into or around the wound defect. As described, this method has demonstrated enhanced wound healing, however, the true therapeutic potential of MSCs appears to be limited due to poor engraftment efficiency and cell retention at the wound site (Freyman et al., 2006). Causes of this phenomenon are still under investigation with evidence suggesting that the hostile wound environment may impede high MSC engraftment in acute wounds. Elevated levels of reactive oxygen species such as those found in ischemic wounds are thought to impede cell engraftment in tissue (Angelos et al., 2006; Yao et al., 2006). In addition, the shear forces generated by the injection process itself may lead to anoikis (Rustad et al., 2012). Alternative delivery systems are therefore being investigated to enhance MSC function within non-healing wounds (Figure 2).

Falanga et al. utilized a fibrin spray system to topically administer autologous MSCs to non-healing lower extremity wounds in human subjects. Stem cells were found to survive within the fibrin layer and migrate into the wound tissue. One subject demonstrated no improvement, four had an average 40% reduction in wound size, and one subject had complete closure of a wound that had previously existed for over 10 years (Falanga et al., 2007).

Building on the idea of providing a scaffolding and external niche from the ischemic tissue, Yoshikawa et al. developed a composite graft combining a commercially available collagen matrix with cultured autologous MSCs. Grafts were applied to 20 patients with intractable dermatopathies due to thermal burns, traumatic wounds, and decubitus ulcers. Thirteen of the 20 wounds demonstrated regeneration of fibrous and fat tissue and underwent re-epithelialization resulting in complete wound closure. Treatment with the composite graft led to sufficient granulation tissue and dermal regeneration to allow for successful skin grafting in an additional five wounds (Yoshikawa et al., 2008).

Hydrogels are synthetic biomaterials that emulate the hygroscopic nature of extracellular matrix making them an ideal vehicle for MSC delivery (Lutolf and Hubbell, 2005). A novel collagen-pullulan hydrogel that is non-cytotoxic and provides protection from oxidative stress was recently described (Wong et al., 2011a). MSCs seeded and cultured in this hydrogel demonstrate significantly greater expression of the stemness genes Oct-4, SOX2, and KLF4 compared to cells plated on standard two-dimensional culture dishes. Secretion of the wound healing and angiogenic cytokines MCP1 and VEGF-A are also found to be increased. Topical hydrogel delivery of MSCs demonstrated significantly accelerated wound closure and improved quality of cutaneous regeneration with greater return of hair follicles and sebaceous glands when compared to intradermal injection strategies. The number of MSCs found within the wound tissue was nearly three times greater at day 7 post-wounding and nearly 10 times greater at day 10 in animals receiving the seeded hydrogen compared to local injection. Co-localization analysis of healed wounds revealed a small percentage (12.5%) of MSCs expressing the endothelial cell marker CD31 representing cells directed toward angiogenesis. The vast majority of engrafted cells differentiated into dermal fibroblasts and pericytes suggesting the wound healing effects were largely secondary to enhanced secretion of paracrine factors. Indeed, levels of VEGF, FGF1, MMP8, and MMP9 were all found to be significantly higher in tissue from wounds treated with MSCs delivered by hydrogel versus intradermal injection (Rustad et al., 2012).

## Heterogeneity in MSC preparations

Despite the data supporting the potential of MSC-based therapy for wound repair, controversy remains. The reported contribution of MSC engraftment to wound repair varies widely in the literature with some authors reporting little to no evidence of cellular engraftment (Duffield et al., 2005; Rustad et al., 2012). The reason for these discrepancies is likely multi-factorial with cell population heterogeneity being a possible contributor. Clonal studies have demonstrated that even with identical isolation and expansion methods, MSC isolation by the traditional plastic adherence technique results in cells that are functionally heterogenous with varying capacities of differentiation (Phinney et al., 1999). Previous studies have also used MSCs from various culture passages, however, investigators have shown that MSCs exhibit different gene expression profiles as they undergo serial passage (Gregory et al., 2005). In addition, MSC function is highly dependent on cues from the culture condition therefore different seeding densities and growth media utilized by investigators add to the heterogeneity of cell preparations used in these studies. These issues speak to the importance of establishing a standardized language when isolating and defining MSCs in the literature and the need for a method of prospective isolation by specific cell surface markers.

## Immunomodulatory properties of MSCs

An important property of MSCs which has been demonstrated both *in vitro* and *in vivo* is the immunosuppressive effect elicited by allogeneic cells. Human MSCs have been shown to suppress CD4+ and CD8+ T-cell proliferation through the secretion of soluble factors including hepatocyte growth factor (Di Nicola et al., 2002) and alter the cytokine secretion profiles of dendritic cells, effector T-cells, and natural killer cells to more anti-inflammatory phenotypes (Aggarwal and Pittenger, 2005). This phenomenon has been exploited to use MSC-therapy to help treat skin graft rejection and graft-versus-host disease (Bartholomew et al., 2002; Le Blanc et al., 2004). How this property may affect the role of MSCs in wound healing has yet to be fully investigated. Some authors have anecdotally theorized that the beneficial effects of MSC therapy on cutaneous repair may in part be due to the prevention or reversal of chronic inflammation. The possible negative side effects of immunosuppression also raise questions that continue to be investigated concerning increased tumorigenicity of cancer cells in animals receiving MSC injections (Djouad et al., 2003). Further work in understanding the systemic effects of MSC are certainly required especially if the use of allogeneic cells is to be considered a clinical possibility.

## Conclusions and future work

The possible benefits of MSC-based therapy in the clinically important area of chronic wounds have been demonstrated in numerous studies. Administration of MSCs has been shown to augment the acute inflammatory response, enhances angiogenesis, accelerates re-epithelialization, and increases wound strength. More importantly, these effects have been observed in clinically relevant conditions of impaired healing such as diabetes.

Although these preliminary findings are promising, several areas require further investigation before large-scale randomized human studies can become feasible. Although the International Society for Cellular Therapy has published minimum criteria to define human MSCs, significant heterogeneity certainly exists within this population (Dominici et al., 2006). Researchers have typically utilized plastic adherence to isolate MSCs in culture, however, macrophages, lymphocytes, endothelial cells, and smooth muscle cells also adhere to plastic and may contaminate early passage populations (Deans and Moseley, 2000). Even in late passage MSCs, cells display morphological and functional heterogeneity (Javazon et al., 2004). Identifying these various subpopulations, understanding their phenotypic properties, and developing methods for prospective isolation by surface marker profiles will be a crucial step in optimizing directed therapy. Also, it remains to be elucidated if the primary contribution of MSCs to cutaneous regeneration is by cellular differentiation or indirectly through paracrine activity. A better understanding of the mechanism of action is needed to develop more efficient treatment strategies. Long-term systemic effects of MSC-therapy have yet to be fully established. Limited data has suggested that the immunosuppressive properties of MSCs could increase susceptibility to malignancies and opportunistic infections (Djouad et al., 2003; Sundin et al., 2006). Additionally, although no instances have been reported in humans, BM-MSCs have been shown to be able to differentiate into carcinoma-associated fibroblasts and sarcomas (Tolar et al., 2007; Mishra et al., 2008). Finally, further investigation into delivery methods specifically designed for the delivery progenitor cells to chronic wounds is necessary to maximize the regenerative properties of MSC-based cell therapy.

### Conflict of interest statement

Geoffrey C. Gurtner is listed on the following patents assigned to Stanford University: (1) Intelligent Biodegradable Pullulan Regenerative Matrix for Tissue Engineering; (2) Efficient stem cell delivery into biomaterials using a novel capillary driven encapsulation technique. Jerry S. Chen and Victor W. Wong have no commercial or financial relationships that could be construed as a potential conflict of interest.
